# Injury Hospitalizations Due to Unintentional Falls among the Aboriginal Population of British Columbia, Canada: Incidence, Changes over Time, and Ecological Analysis of Risk Markers, 1991-2010

**DOI:** 10.1371/journal.pone.0121694

**Published:** 2015-03-20

**Authors:** Andrew Jin, Christopher E. Lalonde, Mariana Brussoni, Rod McCormick, M. Anne George

**Affiliations:** 1 Epidemiology Consultant, Surrey, British Columbia, Canada; 2 Department of Psychology, Faculty of Social Sciences, University of Victoria, Victoria, British Columbia, Canada; 3 Department of Pediatrics, Faculty of Medicine, University of British Columbia, Vancouver, British Columbia, Canada; 4 Child and Family Research Institute, Vancouver, British Columbia, Canada; 5 School of Population and Public Health, University of British Columbia, Vancouver, British Columbia, Canada; 6 Faculty of Human, Social and Educational Development, Thompson Rivers University, Kamloops, British Columbia, Canada; Public Health Agency of Canada, CANADA

## Abstract

**Background:**

Aboriginal people in British Columbia (BC) have higher injury incidence than the general population. Our project describes variability among injury categories, time periods, and geographic, demographic and socio-economic groups. This report focuses on unintentional falls.

**Methods:**

We used BC’s universal health care insurance plan as a population registry, linked to hospital separation and vital statistics databases. We identified Aboriginal people by insurance premium group and birth and death record notations. We identified residents of specific Aboriginal communities by postal code. We calculated crude incidence and Standardized Relative Risk (SRR) of hospitalization for unintentional fall injury, standardized for age, gender and Health Service Delivery Area (HSDA), relative to the total population of BC. We tested hypothesized associations of geographic, socio-economic, and employment-related characteristics with community SRR of injury by linear regression.

**Results:**

During 1991 through 2010, the crude rate of hospitalization for unintentional fall injury in BC was 33.6 per 10,000 person-years. The Aboriginal rate was 49.9 per 10,000 and SRR was 1.89 (95% confidence interval 1.85-1.94). Among those living on reserves SRR was 2.00 (95% CI 1.93-2.07). Northern and non-urban HSDAs had higher SRRs, within both total and Aboriginal populations. In every age and gender category, the HSDA-standardized SRR was higher among the Aboriginal than among the total population. Between 1991 and 2010, crude rates and SRRs declined substantially, but proportionally more among the Aboriginal population, so the gap between the Aboriginal and total population is narrowing, particularly among females and older adults. These community characteristics were associated with higher risk: lower income, lower educational level, worse housing conditions, and more hazardous types of employment.

**Conclusions:**

Over the years, as socio-economic conditions improve, risk of hospitalization due to unintentional fall injury has declined among the Aboriginal population. Women and older adults have benefited more.

## Introduction

Aboriginal people in British Columbia (BC) have higher incidences of injuries than the general population [1–7]. Our project, *Injury in British Columbia’s Aboriginal Communities*: *Building Capacity while Developing Knowledge* [8–11], adds information about variability of incidence rates among injury categories, geographic regions, time periods, and demographic and socio-economic groups within the Aboriginal population. This report focuses on injuries due to unintentional falls.

Studies of the economic burden of unintentional injuries in BC estimated that unintentional falls incident in 2004 cost the province $884 million: $650 million in direct costs (health care) and $235 million of indirect costs (productivity losses), altogether 31% of the $2.81 billion total costs attributed to all injuries. Unintentional falls caused 20% of deaths and 46% of hospitalizations due to all injuries [12]. In 1998, 35% of total costs occurred among persons aged under 25 years, and 25% occurred among the elderly (aged 65 years or older) [13]. The 2004 Special Report of the Provincial Health Officer noted that among the BC population aged 65 years or older, during the period 1992 to 2001, 4.5% of all hospital separations and 11% of all person-days spent in hospital were attributable to injuries from unintentional falls. During the year 2001–2002, among the population aged 65 years or older, 56% of emergency room visits were attributable to injuries from unintentional falls [14]. We think it relevant to measure unintentional falls injury among the Aboriginal population of BC, to help guide development of policies and programs aimed at preventing and treating this condition among the Aboriginal population.

We found one previously published study of the incidence of injury due to unintentional falls among the Aboriginal population of British Columbia [2]. During the period 1992 to 2002 the Age-Standardized Mortality Rate (standardized to the 1991 population of Canada) due to unintentional falls among the Aboriginal population was 1.9 per 10,00 person-years, compared to 0.7 per 10,000 among other people in BC. The incidence of Potential Years of Life Lost (under age 75) was 2.3 per 1,000 person-years among the Aboriginal population, and 0.5 per 1,000 among other people in BC. This study of mortality incidence used the province’s universal health care insurance program as a population registry, and identified Aboriginal people (within the population, and among death records) by record linkage, using a combination of insurance premium group, Indian status, and birth and death record notations. However, left unanswered is the question as to how much of the difference in injury rates between the Aboriginal and general populations is due to the higher proportion of the Aboriginal population who reside in northern, rural or remote locations, where physical environments are more harsh and therefore more conducive to injuries. We adapted this study’s methods, and made three improvements. First, we studied a broader range of injury events (hospitalizations) instead of just deaths, thus enabling more description of variability in incidence rates among geographic regions, demographic groups and time periods. Also, to researchers wishing to estimate economic burden, hospitalizations are more relevant than are deaths. Second, we standardized comparisons of injury rates between the Aboriginal and general populations, by age, gender and also region of the province, thus compensating for the effects of northern location and degree of urbanization. Third, using an ecological approach, where the unit of observation is a geographic unit, we explored associations between standardized incidence of injury due to unintentional falls, and a broad range of hypothesized socio-economic, geographic, and employment-related risk markers.

## Methods

### Ethics review and permission for data access

The University of British Columbia Behavioural Research Ethics Board reviewed and approved our methods (BREB file H06–80585). The Data Stewards representing the BC Ministry of Health and the BC Vital Statistics Agency approved the data access requests. We used existing databases, permanently linked by British Columbia Personal Health Number, maintained by Population Data BC [15–18]. Population Data BC rendered the client records anonymous before our analysis. These data are available on request, from Population Data BC (https://www.popdata.bc.ca/data, contact: Kelly Sanderson, Researcher Liaison Unit Lead, e-mail: kelly.sanderson@popdata.bc.ca; specifications in project file George 11–012) subject to approval by the Data Stewards representing the British Columbia Ministry of Health Services, and the Vital Statistics Agency of British Columbia, for ethical and privacy reasons, because the data pertain to individuals. The data may be accessed and statistically analysed only on Population Data BC’s Secure Research Environment cloud server.

### Population counts

We used the registration and premium billing files [15] of the Medical Services Plan of BC (MSP, the province’s universal health care insurance program) as a population registry, counting the total resident population of BC at the mid-points of fiscal years 1991–1992 through 2009–2010. Within this population, we defined as “Aboriginal” any person with: (a) membership in MSP Premium Group 21 (indicating insurance premiums paid by First Nations and Inuit Health Program, Health Canada, for reason of Indian status, as defined by the Indian Act of Canada), OR (b) one or both parents with Indian status or resident on an Indian Reserve, as indicated on the linked Vital Statistics birth record [16], OR (c) Indian status or resident of an Indian Reserve, as indicated on the linked Vital Statistics death record [17]. We previously described this method, and discussed the quality of the population registry, and validity and limitations of the Aboriginal identification [11]. Our definition of “Aboriginal” is indirectly based on legally recognized Indian status, because for privacy reasons we could not obtain direct access to the Indian Status Registry. However, our indirect method has the advantage of including children who are eligible for Indian status because of their parents’ Indian status, but who have not yet applied to be included in the Indian Status Registry. Family accounts are included in MSP Premium Group 21 if the account’s primary registrant declares Indian status. Some persons with Indian status who are eligible to join MSP Premium Group 21 might not do so because another party (e.g., an employer) pays their premiums. However, this does not happen often because other potential payers have economic incentive to identify people with Indian status so that they can shed the account. A study by the Vital Statistics Agency of BC, using the same method as ours, but with inclusion of additional persons found only in the Indian Status Registry, counted 151,783 Aboriginal persons in BC in 2002 [2], modestly larger than our count of 135,076 persons that year. Since we identified hospitalizations as Aboriginal (or not) by linking to the population registry, the undercounting applies to both the numerator (hospitalization counts) and the denominator (population counts), so there should be no bias in our calculated rates of hospitalization among Aboriginal people.

For purposes of ecologic analysis (see below), within the population we identified Aboriginal “communities” [10]. In BC there are 199 First Nations and Indian Bands recognized by and registered with the government of Canada, inhabiting about 498 reserves. Approximately 44% of the Aboriginal people in BC reside on a reserve and 56% do not. Conceptually, we defined a community as all the Aboriginal people residing on the reserves of one band. Operationally, we assigned Aboriginal people to communities according to postal code of residence. We could thus identify 177 Aboriginal communities in BC. Because Indian reserves do not always have unique and exclusive postal codes, the identified Aboriginal communities include both Aboriginal reserve residents and off-reserve Aboriginal persons living near by, and some communities contain more than one band. This does not perfectly match our conceptual definition, but it is consistent with our underlying intention, to identify culturally homogenous clusters of Aboriginal people living close together.

We aggregated the 177 identified Aboriginal communities to create a subcategory of the Aboriginal population which we called “reserve”. We classified all other Aboriginal persons as “not reserve”.

**Fig 1:**
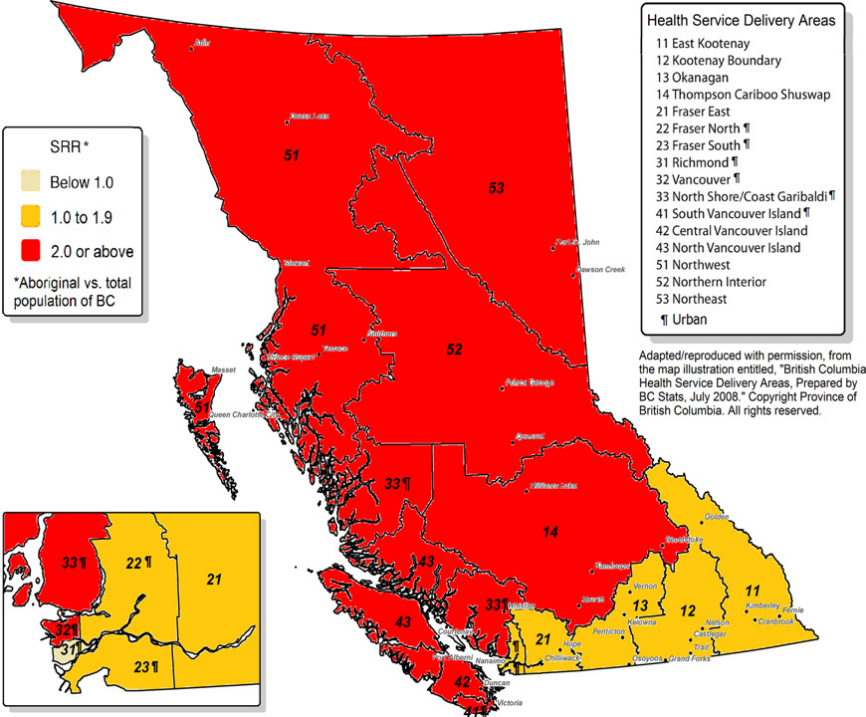
Standardized Relative Risk of hospitalization for unintentional falls injury among Aboriginal populations of Health Service Delivery Areas.

There are sixteen Health Service Delivery Areas (HSDAs) in BC. We classified each HSDA (and its residents) as “urban” or “not urban” according to the proportion of the population of the HSDA residing in an urban centre larger than 100,000 persons [11]. Fig. 1 is a map of BC showing the 16 HSDAs in the province. The six urban HSDAs are marked with the ¶ symbol. The six urban HSDAs comprise metropolitan Vancouver and metropolitan Victoria, and everywhere else in BC is “not urban” by our definition.

We tabulated population counts by fiscal year, gender, 5-year age group, Aboriginal status, community, reserve residence, HSDA, and urban residence.

### Hospitalization counts

We tabulated counts of hospital separations [18] among residents of BC, occurring from April 1, 1991 through March 31, 2010. We considered a hospitalization as “due to injury” if the level of care was “acute” or “rehabilitation”, and the Most Responsible Diagnosis on the discharge record was an International Classification of Diseases Revision 9 (“ICD-9”) numeric code in the range 800 through 999, or an International Classification of Diseases Revision 10 (“ICD-10”) code in the range S00 through T98. We considered an injury as “due to unintentional fall” if the first occurrence of the supplemental injury diagnosis code (indicating intention and external cause) was an ICD-9 E-code in the range E880 through E888, or an ICD-10 code in the range W00 through W19.

Linking individual hospitalization records to the population registry, we tabulated counts of hospitalizations due to unintentional fall injury by calendar year, gender, 5-year age group, Aboriginal status, reserve residence, HSDA, and urban residence.

### Incidence rates of hospitalization

As in previously reported analyses [9–11], we calculated the **crude rate** of hospitalization as the number of hospital separations divided by the person-years of observation (the sum of the annual population counts times the fraction of each year included in the observation period) during the same time period. We considered the crude rate to be a binomial proportion, and we estimated standard errors of the proportion, and 95% confidence intervals of the proportion accordingly. We calculated **Standardized Relative Risk** (SRR) of hospitalization relative to the risk of hospitalization in the reference population (78,256,306 person-years, the combined time-weighted total population of BC from April 1, 1991 through March 31, 2010) using the method of indirect standardization [19], standardizing by gender, 5-year age group, and HSDA, or standardizing for just gender and 5-year age group when calculating SRRs for specific HSDAs. We took the gender, age and HSDA (or gender and age) specific rates of hospitalization in the reference population, and multiplied them by the person-year counts within the corresponding cells of the target population, summing to the indirectly standardized **expected** number of hospitalizations in the target population. We considered the expected rate (the expected number divided by the number of person-years) to be a binomial proportion, and we estimated standard errors and 95% confidence intervals accordingly. The **Standardized Relative Risk** (“SRR”) (relative to the total population of BC) is the crude rate of hospitalizations divided by the expected rate of hospitalizations. Since these rates have the same denominator (person-years) the SRR simplifies to the observed number of hospitalizations divided by the expected number of hospitalizations. This is analogous to the Standardized Mortality Ratio (if death is the event counted), and could also be called the Standardized Incidence Ratio.

We assessed cumulative change in SRR over time as the proportional change between the first and last years of the observation period, i.e., (SRR_2010_/SRR_1991_) −1. To facilitate comparisons, we converted proportional change over the entire period to an annualized change, using this formula.

(SRR2010SRR1991)1/(2010−1991)−1

### Predictors of risk

We studied risk markers for hospitalization due to unintentional fall injury, using an ecological approach, where the unit of observation was the Aboriginal “community” (as defined above). As hypothesized risk markers, we selected socio-economic, housing, and geographic indicators previously developed by Statistics Canada and Aboriginal Affairs and Northern Development Canada. We previously published definitions and reasons for selection of the markers [9,10].

From the 2001 and the 2006 Censuses of Canada, we measured the following hypothesized socio-economic markers of injury risk, for each community: (1) Total (annual) Income per capita, (2) Community Well-Being Income Score (i.e., total annual income per capita, logarithmically scaled) [20], (3) proportion of population, age 25+ years with at least a high school certificate, (4) proportion of population, age 25+ years with university degree, bachelors or higher, (5) average population per room (an index of the degree of crowding in the community’s housing), (6) proportion of dwellings in need of major repairs, (7) proportion of population, age 25+ years, in the labour force, (8) proportion of population, age 25+ years, employed (for pay), (9) proportion of population who identified themselves as “an Aboriginal person, that is, North American Indian, Métis or Inuit (Eskimo)”, (10) proportion of population who gave only one response to the ethnic origin question, and it was a group that could be classified as North American Indian.

We hypothesized that the hazardousness of employment among the community’s labour force would be associated with risk of injury due to falls among the community population, as a direct effect on workers, or indirectly on the broader community population, as an indicator of prevailing socio-economic conditions. Therefore, we calculated the following work-related statistics of injury risk for each community, relative to the population of BC: (11) relative risk of work injury compensation claim, expected from occupational categories, and (12) relative risk of work injury compensation claim, expected from industry categories. We defined these two community-level markers as in a previous report focused on worker compensation injuries [10]. We took the numbers of worker compensation injuries, recorded in BC in 2006, within occupational [21] and industrial [22] categories, then divided by the census labour force population of BC aged 15 years or older in each category. We multiplied these rates of injury by the category-specific census labour force counts within each community, then summed to obtain the community’s expected number of injury claims. We divided this expected number by the community’s total census labour force population, then divided again by the rate of injury claims in BC, with all categories combined.

The Government of Canada’s Department of Aboriginal Affairs and Northern Development has a classification system for calculating funding allocations to First Nation bands [23]. From the tables in this reference, we assigned to communities the following hypothesized geographic markers of injury risk: (13) Remoteness Index (higher score means more remote), and (14) Environmental Index (higher score means more environmentally challenging). These indices are numeric scores, based on geographic latitude, availability of year-round road access, and distance to the nearest “service centre” (a city or town having government services, banks and suppliers).

#### Ecological analysis

For each community, we calculated the age, gender and HSDA-standardized SRR of hospitalization due to unintentional falls injury during the period 1999 through 2003 (a 5-year period centred about the Census year 2001) and during the period 2004 through 2008 (centred about the Census year 2006), relative to the total population of BC during the same time period. Logarithmic transformation approximately normalized the distribution of the SRRs (Kolmogorov-Smirnov statistic 0.048, Shapiro-Wilk statistic 0.990, df = 292, p = 0.048); therefore we used the natural logarithm of SRR as the dependent (Y) variable for regression analysis.

We tested hypotheses of association by performing least-squares linear regressions. We tested census year, hypothesized socio-economic, work-related and geographic markers, in turn as the single independent variable. Variables that had statistically significant association (p<0.05) with SRR of worker compensation injury in univariate analysis were included in subsequent multivariable regression analysis. We used stepwise backwards elimination of variables to arrive at the best-fitting multivariable model. At each step, the variable with the largest p-value was eliminated. Elimination stopped when all independent variables had regression coefficients significantly different from zero (p<0.05).

The regression coefficient (“B”) of each independent variable represents the change in the dependent variable Ln(SRR) that is associated with a unit change in the independent variable. The relative risk associated with a change of one standard deviation (SD) in the independent variable is calculated as the antilogarithm of *B* × *SD*. Repeating the calculation with the lower and upper 95% confidence limits of B gives the confidence limits of the relative risk.

## Results

### Aboriginal status and reserve residence

Table 1 shows crude rates and SRRs of hospitalization for injury due to unintentional falls, during the period 1991–2010, among the total population of BC (i.e., the reference population), the Aboriginal population, the Aboriginal population residing on reserve, and the Aboriginal population residing off-reserve. Table 1 shows a pattern of higher crude incidence among the Aboriginal population than among the total population of BC. The highest crude incidence rate is among the Aboriginal population residing on-reserve. The crude incidence among the Aboriginal population off-reserve is lower, but still higher than among the total population of BC. Standardization by age, gender and HSDA shows the same pattern among the three population groups, but the disparities between the Aboriginal population groups and the total population of BC are magnified.

**Table 1:** Hospital separations for injuries due to unintentional falls [1], British Columbia, 1991–2010 [2].

	P-years [3]	Obs [4]	Exp [5]	Rate [6]	95% CI for Rate			SRR [7]	95% CI for SRR		
BC, total population	78,256,306	262,819	262,818	33.6	33.5	-	33.7	1	[reference]		
BC, Aboriginal	2,541,060	12,683	6,710	49.9	49.1	-	50.8	1.89	1.85	-	1.94
BC, Aboriginal, off-reserve	1,403,813	5,810	3,277	41.4	40.3	-	42.5	1.77	1.71	-	1.83
BC, Aboriginal, on-reserve	1,131,862	6,839	3,421	60.4	59.0	-	61.9	2.00	1.93	-	2.07

Notes:

1. "Injury due to unintentional fall" defined as hospital separation with Most Responsible Diagnosis in the range ICD9:800–999 or ICD10:S00-T98, and supplemental diagnosis in the range ICD9:E880-E888 or ICD10:W00-W19.

2. Injuries occurring during the observation period 1991-Apr-01 to 2010-Mar-31.

3. Person-years is the sum of the annual population counts times the fraction of each year included in the observation period.

4. Observed number of injuries.

5. Expected number, indirectly standardized, based on age, gender and HSDA-specific rates in the total population of BC.

6. Crude Rate per 10,000 person-years.

7. Standardized Relative Risk (compared to the total population of BC) = Observed/Expected.

### HSDAs and urban residence

Table 2 (and S1 Table and S2 Table) show crude rates and age and gender-standardized SRRs of hospitalization for injuries due to unintentional falls, during the period 1991–2010, within the total and Aboriginal populations of the HSDAs. Crude incidence rates and SRRs are highly variable among HSDAs, and this applies among both the Aboriginal population and among the total population of BC. Aggregating the HSDAs into categories of “urban” or “not urban”, one sees that, in general, urban HSDAs have lower crude rates and SRRs than do HSDAs that are not urban, and this applies among both the Aboriginal population and among the total population of BC.

**Table 2:** Hospital separations for injuries due to unintentional falls [1], British Columbia, 1991–2010 [2], by Health Service Delivery Area.

	Total population								Aboriginal population							
HSDA	Rate [3]	95% CI for Rate			SRR [4]	95% CI for SRR			Rate [3]	95% CI for Rate			SRR [4]	95% CI for SRR		
11	45	44	-	47	1.37	1.34	-	1.41	48	41	-	56	1.96	1.57	-	2.45
12	50	49	-	51	1.37	1.33	-	1.40	32	23	-	44	1.39	0.95	-	2.04
13	41	41	-	42	1.02	1.00	-	1.03	38	35	-	41	1.65	1.47	-	1.84
14	43	43	-	44	1.36	1.33	-	1.38	54	51	-	56	2.30	2.14	-	2.46
21	32	31	-	32	0.92	0.91	-	0.94	34	31	-	37	1.55	1.40	-	1.72
22	31	30	-	31	0.98	0.97	-	0.99	33	30	-	37	1.62	1.40	-	1.86
23	29	29	-	29	0.92	0.91	-	0.93	27	24	-	30	1.34	1.17	-	1.54
31	20	20	-	21	0.66	0.64	-	0.67	21	14	-	30	0.97	0.68	-	1.39
32	27	27	-	27	0.80	0.79	-	0.81	43	41	-	46	2.09	1.90	-	2.29
33	36	35	-	36	1.04	1.02	-	1.05	50	47	-	53	2.19	1.99	-	2.41
41	40	40	-	41	0.96	0.95	-	0.97	45	41	-	49	2.08	1.85	-	2.34
42	38	38	-	39	1.03	1.02	-	1.05	50	47	-	53	2.28	2.10	-	2.47
43	40	39	-	41	1.27	1.24	-	1.30	68	64	-	73	3.06	2.73	-	3.44
51	41	40	-	42	1.62	1.57	-	1.67	62	59	-	64	2.57	2.41	-	2.74
52	33	33	-	34	1.30	1.27	-	1.33	61	58	-	64	2.82	2.58	-	3.08
53	29	28	-	30	1.17	1.13	-	1.21	54	49	-	59	2.51	2.16	-	2.91
Urban [5]	30	30	-	31	0.91	0.90	-	0.91	41	40	-	43	1.94	1.85	-	2.04
Not [6]	39	39	-	39	1.15	1.14	-	1.16	54	53	-	55	2.36	2.29	-	2.43
All HSDAs	34	34	-	34	1	[reference]			50	49	-	51	2.24	2.18	-	2.30

Notes:

1. "Injury due to unintentional fall" defined as hospital separation with Most Responsible Diagnosis in the range ICD9:800–999 or ICD10:S00-T98, and supplemental diagnosis in the range ICD9:E880-E888 or ICD10:W00-W19.

2. Injuries occurring during the observation period 1991-Apr-01 to 2010-Mar-31.

3. Crude Rate per 10,000 person-years.

4. Standardized Relative Risk (indirectly standardized by age and gender, compared to the total population of BC) = Observed/Expected.

5. Urban: aggregation of HSDAs 22, 23, 31, 32, 33 and 41, where > 62.3% of the HSDA population live in a large population centre.

6. Not urban: aggregation of HSDAs 11, 12, 13, 14, 21, 42, 43, 51, 52, 53.

Comparing HSDA-specific crude incidence rates, one sees that in most, but not all HSDAs, Aboriginal people have a higher rate of hospitalization for unintentional fall injury than do the total population of BC. Comparing HSDA-specific SRRs, one sees that in every HSDA, standardized for age and gender, Aboriginal people have a higher risk of hospitalization for unintentional fall injury than do the total population. Urban-dwelling Aboriginal people have a higher risk of hospitalization for unintentional fall injury than do the urban dwelling total population of BC. Aboriginal people residing in non-urban HSDAS also have a higher risk of hospitalization for unintentional fall injury than do the total population of BC residing in non-urban HSDAs.

### Age and gender

Table 3 (and S3 Table and S4 Table) show crude rates and age and gender-specific, HSDA-standardized SRRs of hospitalization for injuries due to unintentional falls, during the period 1991–2010, within specific age and gender categories of the total and Aboriginal populations of BC. Among those aged less than 50 years, males have higher incidence rates of hospitalization for unintentional fall injury than do females, but among those aged 50 years and older, females have higher incidence rates, and the excess among females is even larger among persons aged 70 years or older. This pattern applies both among the total population and among the Aboriginal population of BC. Among the total population of BC, the incidence rate is lowest in the 20 to 29 years of age group, and increases with age, this pattern occurs both among males and among females. Among the Aboriginal population of BC, the incidence rate is lowest in the 10 to 19 years of age group, and increases with age, this pattern occurs both among males and among females.

**Table 3:** Hospital separations for injuries due to unintentional falls [1], British Columbia, 1991–2010 [2], by gender and age.

		Total population					Aboriginal population							
Gender	Age	Rate [3]	95% CI for Rate			SRR [ref]	Rate [3]	95% CI for Rate			SRR [4]	95% CI for SRR		
F	0–9	17	17	-	18	1	29	27	-	31	1.51	1.39	-	1.65
F	10–19	10	10	-	10	1	21	19	-	23	1.67	1.49	-	1.88
F	20–29	7	7	-	8	1	26	24	-	29	2.65	2.31	-	3.04
F	30–39	10	9	-	10	1	35	33	-	38	2.78	2.47	-	3.14
F	40–49	14	13	-	14	1	46	43	-	50	2.76	2.46	-	3.10
F	50–59	26	26	-	27	1	91	85	-	97	2.86	2.56	-	3.19
F	60–69	49	48	-	49	1	152	142	-	162	2.50	2.25	-	2.78
F	70–79	119	117	-	120	1	283	264	-	303	2.05	1.86	-	2.27
F	80+	355	352	-	358	1	481	447	-	517	1.17	1.08	-	1.27
M	0–9	23	22	-	23	1	38	35	-	40	1.54	1.43	-	1.66
M	10–19	24	24	-	25	1	35	33	-	38	1.25	1.16	-	1.35
M	20–29	17	17	-	17	1	36	33	-	39	1.73	1.57	-	1.91
M	30–39	17	17	-	17	1	42	39	-	45	1.99	1.81	-	2.19
M	40–49	19	19	-	20	1	52	48	-	55	2.18	1.97	-	2.41
M	50–59	24	24	-	24	1	70	65	-	75	2.46	2.18	-	2.78
M	60–69	34	34	-	35	1	109	100	-	118	2.52	2.20	-	2.88
M	70–79	64	63	-	65	1	169	153	-	187	2.27	1.95	-	2.64
M	80+	186	184	-	189	1	241	213	-	273	1.14	1.00	-	1.30

Notes:

1. "Injury due to unintentional fall" defined as hospital separation with Most Responsible Diagnosis in the range ICD9:800–999 or ICD10:S00-T98, and supplemental diagnosis in the range ICD9:E880-E888 or ICD10:W00-W19.

2. Injuries occurring during the observation period 1991-Apr-01 to 2010-Mar-31.

3. Crude Rate per 10,000 person-years.

4. Standardized Relative Risk (indirectly standardized by age, gender and HSDA, compared to the total population of BC) = Observed/Expected.

Comparing age and gender-specific crude incidence rates, one sees that all age and gender categories, Aboriginal people have a higher rate of hospitalization for unintentional fall injury than do the total population of BC. Comparing age and gender-specific, HSDA-standardized SRRs, one sees that in every age and gender category, Aboriginal people have a higher risk of hospitalization for unintentional fall injury than do the total population of BC. The increased relative risk among Aboriginal people is less severe among the young (less than 20 years of age) and the very elderly (80 years or older).

### Changes over time

**Fig 2:**
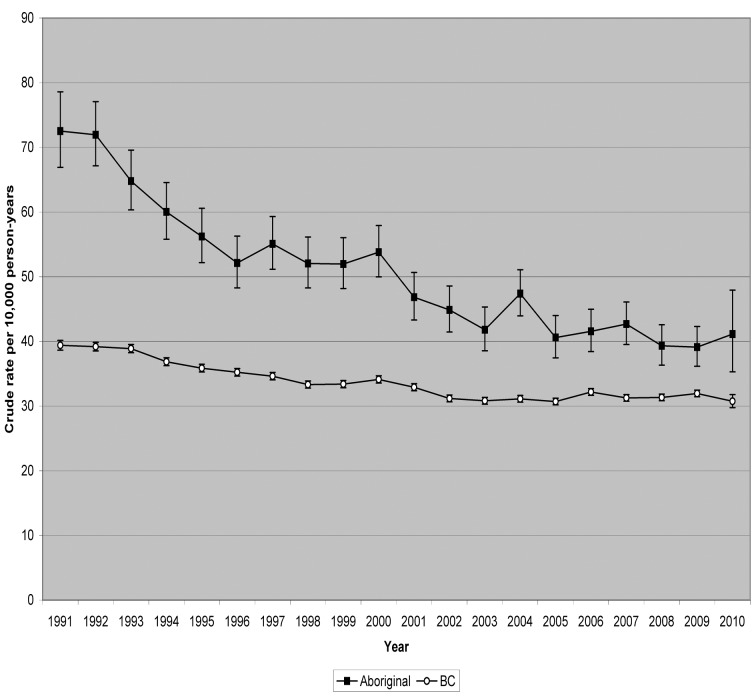
Hospitalizations due to unintentional falls, British Columbia, 1991–2010, crude rate by year.

**Fig 3:**
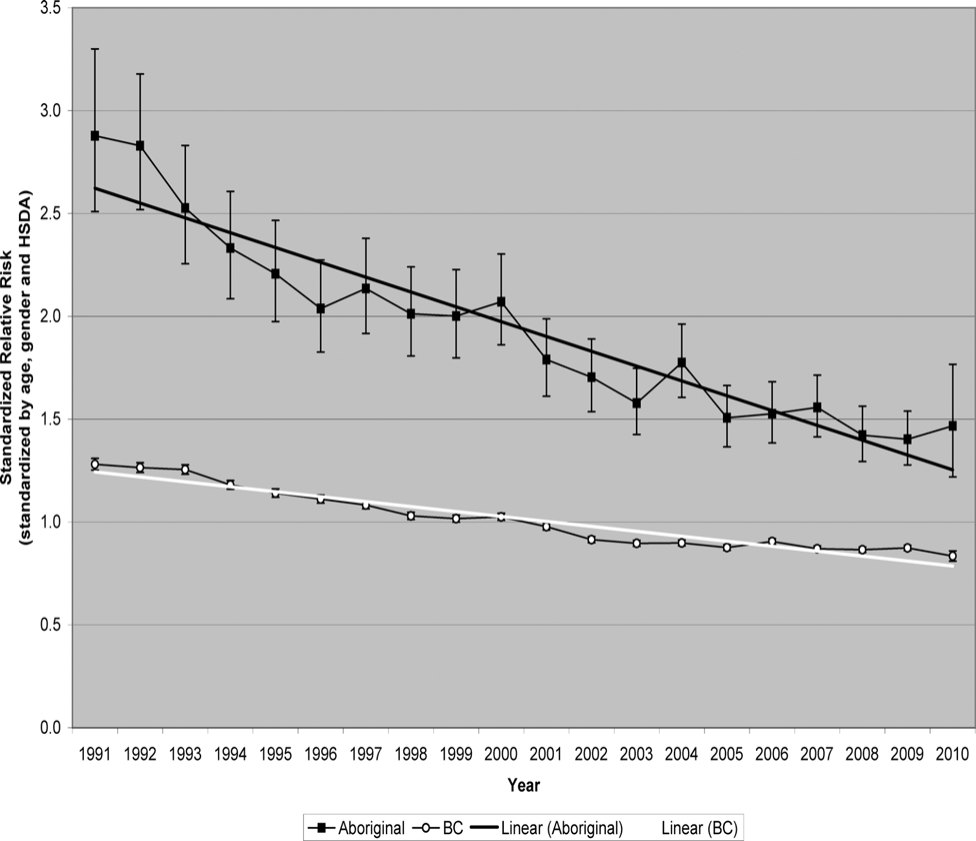
Hospitalizations due to unintentional falls, British Columbia, 1991–2010, standardized relative risk by year.

Tables 4 and 5 (and S5 Table and S6 Table) show crude rates and SRRs of hospitalizations from injuries due to unintentional falls, during the period 1991–2010, among the total and Aboriginal populations of BC (Table 4), and demographic categories within these populations (Table 5), by year. SRRs have been standardized for age, gender, and HSDA. Recall that the reference population is the combined total population of BC during the entire period (1991 through 2010). Thus, the SRR for the total population of BC in a particular year can be higher or lower than one, but the average of the SRRs for the total population of BC, over all the years, will be one. Table 4, and Figs. 2 and 3, show that in every year, the Aboriginal population had a higher crude rate and a higher SRR than did the total population of BC, but over the period 1991–2010, reductions in crude rates and standardized risks have been sustained, and substantial, among both populations. Table 5 shows that reductions have also been substantial among males, females, and youth (age under 25 years) within both the Aboriginal and total populations of BC. Standardized by age, the reductions in SRR are independent of any changes over time in the age distributions of the populations. Among the Aboriginal population of BC, the proportion residing in urban HSDAs increased from 28.7% in 1991 to 30.1% in 2010. Among the total population of BC, the proportion increased from 60.0% in 1991 to 62.4% in 2010. Having standardized by HSDA, we can say that the reductions in SRR are independent of any effect from the increasing concentration of the populations into urban areas.

**Table 4:** Hospital separations for injuries due to unintentional falls [1], British Columbia, 1991–2010 [2], by calendar year.

	Total population								Aboriginal population							
Year	Rate [3]	95% CI for Rate			SRR [4]	95% CI for SRR			Rate [3]	95% CI for Rate			SRR [4]	95% CI for SRR		
1991	39	39	-	40	1.28	1.25	-	1.31	73	67	-	79	2.88	2.51	-	3.30
1992	39	39	-	40	1.26	1.24	-	1.29	72	67	-	77	2.83	2.52	-	3.18
1993	39	38	-	40	1.26	1.23	-	1.28	65	60	-	70	2.53	2.26	-	2.83
1994	37	36	-	37	1.18	1.16	-	1.20	60	56	-	65	2.33	2.09	-	2.61
1995	36	35	-	36	1.14	1.12	-	1.16	56	52	-	61	2.21	1.97	-	2.47
1996	35	35	-	36	1.11	1.09	-	1.13	52	48	-	56	2.04	1.83	-	2.27
1997	35	34	-	35	1.08	1.06	-	1.10	55	51	-	59	2.14	1.92	-	2.38
1998	33	33	-	34	1.03	1.01	-	1.05	52	48	-	56	2.01	1.81	-	2.24
1999	33	33	-	34	1.02	1.00	-	1.03	52	48	-	56	2.00	1.80	-	2.23
2000	34	34	-	35	1.03	1.01	-	1.04	54	50	-	58	2.07	1.86	-	2.30
2001	33	32	-	33	0.98	0.96	-	0.99	47	43	-	51	1.79	1.61	-	1.99
2002	31	31	-	32	0.91	0.90	-	0.93	45	41	-	49	1.70	1.54	-	1.89
2003	31	30	-	31	0.90	0.88	-	0.91	42	39	-	45	1.58	1.43	-	1.75
2004	31	31	-	32	0.90	0.88	-	0.91	47	44	-	51	1.78	1.61	-	1.96
2005	31	30	-	31	0.88	0.86	-	0.89	41	37	-	44	1.51	1.37	-	1.66
2006	32	32	-	33	0.91	0.89	-	0.92	42	38	-	45	1.53	1.38	-	1.68
2007	31	31	-	32	0.87	0.86	-	0.88	43	40	-	46	1.56	1.41	-	1.71
2008	31	31	-	32	0.87	0.85	-	0.88	39	36	-	43	1.42	1.29	-	1.56
2009	32	31	-	32	0.87	0.86	-	0.89	39	36	-	42	1.40	1.28	-	1.54
2010	31	30	-	32	0.83	0.81	-	0.86	41	35	-	48	1.47	1.22	-	1.77
1991–2010	34	33	-	34	1	[reference]			50	49	-	51	1.89	1.85	-	1.94

Notes:

1. "Injury due to unintentional fall" defined as hospital separation with Most Responsible Diagnosis in the range ICD9:800–999 or ICD10:S00-T98, and supplemental diagnosis in the range ICD9:E880-E888 or ICD10:W00-W19.

2. Injuries occurring during the observation period 1991-Apr-01 to 2010-Mar-31.

3. Crude Rate per 10,000 person-years.

4. Standardized Relative Risk (indirectly standardized by age, gender and HSDA, compared to the total population of BC, 1991 to 2010) = Observed/Expected.

**Table 5:** Hospital separations for injury due to unintentional fall [1], British Columbia, 1991–2010 [2], by calendar year and demographic category.

Demographic category	Year	Person-years[3]	Obs [4]	Exp [5]	Rate [6]	95% CI for Rate			SRR [7]	95% CI for SRR		
Aboriginal	1991	81,353	590	205	73	67	-	79	2.88	2.51	-	3.30
Aboriginal	2010	39,871	164	112	41	35	-	48	1.47	1.22	-	1.77
Aboriginal, Male	1991	39,605	284	107	72	64	-	81	2.64	2.19	-	3.19
Aboriginal, Male	2010	19,739	89	55	45	37	-	55	1.62	1.24	-	2.11
Aboriginal, Female	1991	41,281	298	93	72	64	-	81	3.21	2.62	-	3.93
Aboriginal, Female	2010	20,052	75	56	37	30	-	47	1.35	1.04	-	1.76
Aboriginal, age under 25 years	1991	41,961	231	85	55	48	-	63	2.71	2.19	-	3.35
Aboriginal, age under 25 years	2010	18,972	36	38	19	14	-	26	0.94	0.69	-	1.30
BC	1991	2,566,094	10,109	7,894	39	39	-	40	1.28	1.25	-	1.31
BC	2010	1,158,039	3,561	4,268	31	30	-	32	0.83	0.81	-	0.86
BC, Male	1991	1,261,517	4,527	3,428	36	35	-	37	1.32	1.28	-	1.37
BC, Male	2010	573,085	1,412	1,738	25	23	-	26	0.81	0.78	-	0.85
BC, Female	1991	1,298,489	5,459	4,379	42	41	-	43	1.25	1.21	-	1.28
BC, Female	2010	584,475	2,147	2,523	37	35	-	38	0.85	0.82	-	0.88
BC, age under 25 years	1991	880,765	2,501	1,565	28	27	-	30	1.60	1.52	-	1.68
BC, age under 25 years	2010	330,130	345	557	10	9	-	12	0.62	0.57	-	0.67
BC	1991–2010	78,256,306	262,819	262,693	34	33	-	34	1	[reference]		
BC, Male	1991–2010	38,715,563	110,420	110,420	29	28	-	29	1	[reference, male]		
BC, Female	1991–2010	39,474,907	151,295	151,294	38	38	-	39	1	[reference, female]		
BC, age under 25 years	1991–2010	24,727,465	43,114	43,113	17	17	-	18	1	[reference, under 25]		

Notes:

1. "Injury due to unintentional fall" defined as Most Responsible Diagnosis in the range ICD9:800–999 or ICD10:S00-T98, and supplemental diagnosis in the range ICD9:E880-E888 or ICD10:W00-W19.

2. Separations occurring during the observation period 1991-Apr-01 to 2010-Mar-31.

3. Person-years is the annual population count times the fraction of the year included in the observation period.

4. Observed number of hospital separations (acute or rehabilitation care).

5. Expected number, indirectly standardized, based on age, gender and HSDA-specific rates in the total population of BC during the entire observation period.

6. Crude Rate per 10,000 person-years.

7. Standardized Relative Risk (compared to the total population of BC during the same observation period) = Observed/Expected.

Table 6 shows proportional changes in SRR between 1991 and 2010, among the Aboriginal and total populations of BC, and demographic categories within these populations As we described in an earlier report comparing time trends among various injury categories [11], between 1991 and 2010 there was 49.0% decline in SRR of hospitalization due to unintentional falls among the Aboriginal population (annualized change of −3.5%, 95% confidence interval: −4.6% to −2.3%), compared to 34.8% decline among the total population of BC (annualized change of −2.2%, 95% CI −2.4% to −2.0%). The disparity between the Aboriginal and total populations was statistically significant (p = 0.039, 2-sided). Among Aboriginal females there was 57.9% decline in SRR, substantially more than the 31.7% decline among females in the total population of BC (p = 0.005, 2-sided). But among Aboriginal males, and among Aboriginal youth (age under 25 years), the declines in SRR were practically same as the declines among the same demographic groups within the total population of BC. The greater decline in SRR means that the gap between the Aboriginal and total populations of BC is shrinking, but the comparisons among demographic categories indicates that the closing gap is occurring due to more rapid improvements among females and older adults.

**Table 6:** Standardized Relative Risks of hospitalization for injury due to unintentional fall, British Columbia, 1991–2010.

Demographic category	SRR 1991	SRR 2010	1991 to 2010% change	p*	Annual % change	L95CL	U95CL
Aboriginal	2.88	1.47	-49.0%	0.039	-3.5%	-4.6%	-2.3%
Aboriginal, Male	2.64	1.62	-38.9%	0.969	-2.6%	-4.2%	-0.9%
Aboriginal, Female	3.21	1.35	-57.9%	0.005	-4.5%	-6.1%	-2.8%
Aboriginal, age under 25 years	2.71	0.94	-65.2%	0.592	-5.4%	-7.3%	-3.5%
BC	1.28	0.83	-34.8%		-2.2%	-2.4%	-2.0%
BC, Male	1.32	0.81	-38.5%		-2.5%	-2.8%	-2.2%
BC, Female	1.25	0.85	-31.7%		-2.0%	-2.2%	-1.7%
BC, age under 25 years	1.60	0.62	-61.2%		-4.9%	-5.3%	-4.4%

Notes:

* probability (2-sided, z-test) that Ln((SRR 2010)/(SRR 1991)) Aboriginal = Ln((SRR 2010)/(SRR 1991)) BC

SRR: Standardized Relative Risk (indirectly standardized by age, gender and HSDA, compared to the total population of BC, 1991 to 2010) = Observed/Expected

L95CL: lower limit of the 95% confidence interval for the annualized % change.

U95CL: upper limit of the 95% confidence interval for the annualized % change.

### Ecological analysis of predictors of risk

Table 7 shows statistics from regression models with a single independent (X) variable. We identified the following variables as statistically significant (p<0.05 for the null hypothesis that the regression coefficient “B” is zero) predictors of a community’s injury hospitalization risk: (1) annual income per capita (measured in $): increase of one standard deviation ($5,900) was associated with a reduction in SRR of hospitalization from injury due to unintentional fall by a factor of 0.915; (2) annual income per capita (measured on a logarithmic scale): increase of one standard deviation was associated with a reduction in SRR by a factor of 0.909; (3) proportion of the adult population who had completed high school: increase of one standard deviation (17.4%) was associated with a reduction in SRR by a factor of 0.908; (4) proportion of dwellings in need of major repair: increase of one standard deviation (19.2%) was associated with an increase in SRR by a factor of 1.089; (5) relative risk of worker compensation claim based on the occupational distribution of the community’s labour force: increase of one standard deviation (0.36) was associated with an increase in SRR by a factor of 1.087; (6) relative risk of worker compensation claim based on the industry distribution of the community’s labour force: increase of one standard deviation (0.34) was associated with an increase in SRR by a factor of 1.106.

**Table 7:** Ecologic analysis of risk of hospitalization due to unintentional falls injury among BC First Nations communities, 1999–2008; regression [1] statistics from models with one independent (X) variable.

X Variable	units	min	max	mean [2]	SD [2]	N	R^2^	B[3]	SE[4]	p[5]	RR Ratio per SD [6]	L95CL [7]	U95CL [8]
Census	1 year	2001	2006	2003.5	2.5	292	0.007	-0.017	0.011	0.147	0.959	0.907	1.015
IncomePerCapita1000	$1,000	5.3	50.9	13.1	5.9	139	0.030	-0.015	0.007	0.042	0.915	0.841	0.997
IncomeScore	1	32.6	108.1	60.2	12.7	139	0.041	-0.008	0.003	0.017	0.909	0.841	0.983
HighSchool	1%	0.0	116.7	55.7	17.4	240	0.032	-0.006	0.002	0.005	0.908	0.848	0.971
UniversityDegree	1%	0.0	34.3	3.9	5.8	240	0.009	-0.008	0.005	0.133	0.954	0.897	1.015
PopPerRoom	1	0.30	1.11	0.53	0.11	240	0.016	0.644	0.327	0.050	1.074	1.000	1.154
NeedMajorRepairs	1%	0.0	120.0	32.7	19.2	240	0.022	0.004	0.002	0.022	1.089	1.013	1.172
LabourForce	1%	9.9	100.0	61.7	12.3	240	0.000	0.000	0.003	0.965	0.998	0.926	1.077
Employed	1%	7.7	77.3	47.3	11.0	240	0.004	-0.003	0.003	0.312	0.964	0.897	1.035
OccupationRisk	RR	0.00	2.71	1.12	0.36	240	0.018	0.234	0.113	0.040	1.087	1.004	1.177
IndustryRisk	RR	0.00	3.92	1.11	0.34	240	0.019	0.300	0.139	0.032	1.106	1.009	1.212
Remoteness	1	0.08	1.35	0.23	0.22	290	0.003	0.111	0.123	0.367	1.024	0.972	1.080
EnvironIndex	1	0.40	3.00	0.65	0.38	290	0.000	0.015	0.074	0.842	1.006	0.951	1.064
Aboriginal	1%	5.7	100.0	84.7	23.2	240	0.014	0.002	0.001	0.069	1.058	0.996	1.124
NAIndian	1%	5.6	103.1	81.5	23.8	240	0.009	0.002	0.001	0.138	1.048	0.985	1.114

Notes:

1. The dependent (Y) variable is Ln(SRR of hospitalization due to unintentional falls injury), and regression is weighted by person-years.

2. Unweighted mean and standard deviation (SD) of the independent (X) variable

3. B = regression coefficient

4. SE = standard error of the regression coefficient

5. p = probability that B = 0

6. Relative Risk Ratio per SD = exp(BxSD). One SD change in the independent variable is associated with a change in the Standardized Relative Risk of injury by this factor. E.g., one SD change in Income Per Capita ($5,900) is associated with reduction is SRR by a factor of 0.915.

7. Lower limit of the 95% confidence interval for the Relative Risk Ratio per SD.

8. Upper limit of the 95% confidence interval for the Relative Risk Ratio per SD.

After step-wise regression, one independent variable remained in the best-fitting model: the proportion of the adult population who had completed high school (p = 0.005). We did not find any multivariable model in which all of the independent variables were statistically significantly associated with SRR of hospitalization from unintentional fall injury.

## Discussion

Aboriginal people have higher incidence of hospitalization due to unintentional falls than the total population. Standardizing for age increases the disparity, because the Aboriginal population are on average younger than the total population of BC, and hospitalization for unintentional falls injury is most frequent among the elderly. Standardizing for geographic area of residence (HSDA) reduces the disparity, because the Aboriginal population are more likely to reside in northern or non-urban HSDAs, where hospitalization for unintentional falls injury occurs more frequently. Standardizing for both age and HSDA, Aboriginal people have about twice the risk of hospitalization due to unintentional falls injury as do the total population of BC. The disparity is greater comparing Aboriginal people living on-reserve to the total population of BC, than comparing Aboriginal people living off-reserve to the total population of BC. Having standardized for HSDA, we can say that this difference is not simply because most reserve communities are located in northern or non-urbanized areas, where unintentional falls injuries occur more frequently, there may be less contention for hospital beds, and physicians may be more likely to admit injured persons to hospital because of a lack of outpatient or community-care options. There must be other factors related to living on a reserve, indeed to the Aboriginal condition, that increase risk of unintentional falls injury. Whatever these factors may be, they are historically long-standing, but they are diminishing with time. Changes over time in risk of hospitalization from unintentional falls injury suggest that the gap between the Aboriginal population and the total population of BC is narrowing.

We found an interesting pattern with respect to age, gender and time. Among people aged 50 years and over, risk of hospitalization due to unintentional fall injury is higher among females than males, but among those under 50 years, the risk is higher among males. Among females and adults over 25 years of age, risk is declining faster among the Aboriginal population than among the general population of BC, but among males and youth under 25 years, risk is declining at the same rate as among the general population. One might hypothesize that unintentional fall injuries among women and older adults are mostly related to osteoporosis and other age-related physical frailties, whereas fall injuries among males and youth are more strongly influenced by behaviours such as employment, recreational activities and substance use. The hospital discharge record can have as many as 25 diagnostic codes (maximum 16 prior to 2001), but the present analysis used just two: the most responsible diagnosis, and the first occurrence of an external cause of injury code. Further analysis of additional diagnostic codes may help elucidate factors contributing to the fall injury.

Our ecological analysis of hypothesized socio-economic, employment-related, and geographic risk markers may help explain disparities of risk among Aboriginal reserve communities. We observed that lower income, lower educational level, worse housing conditions and more hazardous types of employment are associated with increased risk of hospitalization from unintentional falls injury. All of these factors could be classified as socio-economic disadvantages. Multivariable regression analyses, intended to elucidate the relative importance of these factors, their relationships and the pathways by which they might exert their effects were not informative, because we encountered problems of low statistical power. First, hospitalization from unintentional falls injury is a very narrow category of injury (crude incidence among the on-reserve Aboriginal population is 60.4 per 10,000 per year), and with the small communities we studied (mean population 351, SD 419), the expected number of injury events per 5-year observation period is small (11). Consequently, the rate of hospitalization in a community, which is a proportion, will have a very high standard error, simply on the basis of random sampling variability. The ecological multivariable analysis method worked better with broader, more frequent outcome categories, for example, hospitalizations from all injuries combined (crude incidence 252 per 10,000 per year [9]), or worker compensation injury claims with all injury categories combined (crude incidence 105 per 10,000 per year [10]). Second, the ecological characteristics of the communities were derived mostly from custom tabulations of the Census of Canada, aggregating data pertaining to the populations of specific reserves. Census geography is more precise than postal codes, so these data pertain only to residents of reserves. On the other hand, they include all residents of the reserves, even those who do not meet our definition of “Aboriginal”. Thus, the community population among whom we calculated injury risks is not exactly the same as the community population among whom we ascertained the ecological predictors of risk. This mismatch probably increased the component of random variability in our regression models, thus making the coefficient of determination (R^2^) smaller, and making it less likely that we would find statistically significant associations between injury risk and the hypothesized ecological predictors of risk. But this is all the more reason to take seriously the associations that we did find.

A limitation of all ecological studies is the fallacy that associations observed at the community level apply also at the individual level. For example, we found that a preponderance of more hazardous types of employment among the community’s labour force is associated with increased risk of hospitalization due to unintentional falls among the community’s population. It is plausible that individuals employed in hazardous occupations or industries would be more likely to suffer injury from falls. But it is also possible that more hazardous employment is an indicator of a higher prevalence of socio-economic disadvantage among the community’s population, and the injurious consequences are borne mostly by those in the community who are not employed at all.

Our ecological analysis of risk markers did not include off-reserve Aboriginal or non-Aboriginal communities. Therefore, the findings say nothing about disparities of unintentional fall injury risk between the on-reserve and off-reserve Aboriginal populations, or between the Aboriginal and total populations of BC. Those questions invite further research, and will be explored in a future report.

In this study we did not count injuries, we counted hospitalizations due to injury. Hospitalizations indicate injury burden, but are also influenced by utilization factors such as availability of hospital beds, outpatient and community care options, and patterns of medical practice. These factors vary among regions of the province, and we standardized risks by HSDA, so the comparison between the Aboriginal and the total populations of BC should not be biased. However, acting within HSDAs there may be other factors that affect Aboriginal people differently from the general population. Hospitalizations do not include all injuries: excluded are milder injuries, and also extremely severe injuries resulting in immediate death. Some injuries may lead to more than one hospitalization. Hospitalizations [9,11], worker compensation claims [10], primary care visits (the subject of a future report), and deaths [2–7] are pieces of a larger picture.

The magnitude of the disparity in risk of hospitalization due to unintentional falls (SRR of 1.89) was somewhat less than the disparity in risk of death. During the period 1991 to 2002, people with Indian status residing on-reserve experienced 2.85 times more, and those residing off-reserve experienced 1.76 times more Potential Years of Life Lost (PYLL) due to unintentional falls than did other Canadians [7]. During the period 1992 to 2002, people with Indian status experienced an Age Standardized Mortality Rate due to unintentional falls 2.7 times higher, and suffered 4.6 times more PYLL than other people in BC [2]. Aboriginal people may have more severe falls than the general population. Alternatively, Aboriginal people may under-utilize health care services, relative to the severity of their falls. But in general, our findings are consistent with the mortality experience. This suggests that our observations reflect real disparities and changes in the underlying incidence of unintentional falls injury, and are not merely artefacts related to hospital utilization.

In this analysis, the main outcome of interest was SRR of hospitalization (standardized by age, gender and HSDA), because this was useful for testing hypotheses of association with Aboriginal ethnicity, reserve residence, urban residence and time period. However, the crude rate reflects the true burden of injury. Further analysis, incorporating time series modelling and forecasting of rates, population and demographic forecasting, and the resource-intensity of hospitalization categories could provide more information about the burden of unintentional falls injury among our target population.

## Conclusions

Aboriginal people need culturally appropriate programs to mitigate unintentional falls injury even more than the general population do. As socio-economic conditions improve [24], risk of unintentional fall injury has declined among the Aboriginal population. Females and older adults seem to have benefited more. To improve health and safety, and to close the gap between the Aboriginal and the general populations require that we also address the socio-economic determinants of health [25].

## Supporting Information

S1 Table Hospital separations for injuries due to unintentional falls [1], British Columbia, 1991–2010 [2], by Health Service Delivery Area. (DOC)

S2 Table Hospital separations for injuries due to unintentional falls [1], Aboriginal BC, 1991–2010 [2], by Health Service Delivery Area. (DOC)

S3 Table Hospital separations for injuries due to unintentional falls [1], British Columbia, 1991–2010 [2], by gender and age. (DOC)

S4 Table Hospital separations for injuries due to unintentional falls [1], Aboriginal BC, 1991–2010 [2], by gender and age. (DOC)

S5 Table Hospital separations for injuries due to unintentional falls [1], British Columbia, 1991–2010 [2], by calendar year. (DOC)

S6 Table Hospital separations for injuries due to unintentional falls [1], Aboriginal BC, 1991–2010 [2], by calendar year. (DOC)
