# Supplementary material for: Injury Hospitalizations Due to Unintentional Falls among the Aboriginal Population of British Columbia, Canada: Incidence, Changes over Time, and Ecological Analysis of Risk Markers, 1991-2010
Source: PLoS One. 2015 Mar 20;10(3):e0121694. doi: 10.1371/journal.pone.0121694 (PMC4368097; doi:10.1371/journal.pone.0121694)
Supplement: S1 Table — (DOC) [file pone.0121694.s001.doc]

| **S1 Table: Hospital separations for injuries due to unintentional falls [1], British Columbia, 1991-2010 [2], by Health Service Delivery Area** | | | | | | | | | | | |
| --- | --- | --- | --- | --- | --- | --- | --- | --- | --- | --- | --- |
|  |  |  |  |  |  |  |  |  |  |  |  |
| **HSDA** | **P-years [3]** | **Obs [4]** | **Exp [5]** | **Rate [6]** | **95% CI for Rate** | | | **SRR [7]** | **95% CI for SRR** | | |
|  |  |  |  |  |  |  |  |  |  |  |  |
| 11 | 1,494,122 | 6,796 | 4,950 | 45 | 44 | - | 47 | 1.37 | 1.34 | - | 1.41 |
| 12 | 1,520,070 | 7,639 | 5,581 | 50 | 49 | - | 51 | 1.37 | 1.33 | - | 1.40 |
| 13 | 5,916,943 | 24,476 | 24,070 | 41 | 41 | - | 42 | 1.02 | 1.00 | - | 1.03 |
| 14 | 4,061,010 | 17,534 | 12,918 | 43 | 43 | - | 44 | 1.36 | 1.33 | - | 1.38 |
| 21 | 4,555,474 | 14,399 | 15,625 | 32 | 31 | - | 32 | 0.92 | 0.91 | - | 0.94 |
| 22 | 9,958,993 | 30,433 | 30,968 | 31 | 30 | - | 31 | 0.98 | 0.97 | - | 0.99 |
| 23 | 11,161,270 | 32,354 | 35,169 | 29 | 29 | - | 29 | 0.92 | 0.91 | - | 0.93 |
| 31 | 3,319,052 | 6,777 | 10,339 | 20 | 20 | - | 21 | 0.66 | 0.64 | - | 0.67 |
| 32 | 11,335,213 | 30,621 | 38,242 | 27 | 27 | - | 27 | 0.80 | 0.79 | - | 0.81 |
| 33 | 4,994,095 | 17,949 | 17,290 | 36 | 35 | - | 36 | 1.04 | 1.02 | - | 1.05 |
| 41 | 6,396,003 | 25,630 | 26,662 | 40 | 40 | - | 41 | 0.96 | 0.95 | - | 0.97 |
| 42 | 4,555,818 | 17,519 | 16,947 | 38 | 38 | - | 39 | 1.03 | 1.02 | - | 1.05 |
| 43 | 2,181,505 | 8,742 | 6,866 | 40 | 39 | - | 41 | 1.27 | 1.24 | - | 1.30 |
| 51 | 1,617,996 | 6,649 | 4,103 | 41 | 40 | - | 42 | 1.62 | 1.57 | - | 1.67 |
| 52 | 2,865,914 | 9,561 | 7,356 | 33 | 33 | - | 34 | 1.30 | 1.27 | - | 1.33 |
| 53 | 1,253,604 | 3,626 | 3,101 | 29 | 28 | - | 30 | 1.17 | 1.13 | - | 1.21 |
|  |  |  |  |  |  |  |  |  |  |  |  |
| Urban [8] | 47,164,624 | 143,764 | 158,670 | 30 | 30 | - | 31 | 0.91 | 0.90 | - | 0.91 |
| Not [9] | 30,022,455 | 116,941 | 101,518 | 39 | 39 | - | 39 | 1.15 | 1.14 | - | 1.16 |
|  |  |  |  |  |  |  |  |  |  |  |  |
| All HSDAs | 77,187,080 | 260,705 | 260,188 | 34 | 34 | - | 34 | 1 | [reference] | | |
|  |  |  |  |  |  |  |  |  |  |  |  |

| **Notes:** |
| --- |
| 1. "Injury due to unintentional fall" defined as hospital separation with Most Responsible Diagnosis in the range ICD9:800-999 or |
| ICD10:S00-T98, and supplemental diagnosis in the range ICD9:E880-E888 or ICD10:W00-W19. |
| 2. Injuries occurring during the observation period 1991-Apr-01 to 2010-Mar-31. |
| 3. Person-years is the sum of the annual population counts times the fraction of each year included in the observation period. |
| 4. Observed number of injuries. |
| 5. Expected number, indirectly standardized, based on age and gender-specific rates in the total population of BC. |
| 6. Crude Rate per 10,000 person-years. |
| 7. Standardized Relative Risk (compared to the total population of BC) = Observed/Expected. |
| 8. Urban: aggregation of HSDAs 22, 23, 31, 32, 33 and 41, where > 62.3% of the HSDA population live in a large population centre. |
| 9. Not urban: aggregation of HSDAs 11, 12, 13, 14, 21, 42, 43, 51, 52, 53. |
