# Supplementary material for: Injury Hospitalizations Due to Unintentional Falls among the Aboriginal Population of British Columbia, Canada: Incidence, Changes over Time, and Ecological Analysis of Risk Markers, 1991-2010
Source: PLoS One. 2015 Mar 20;10(3):e0121694. doi: 10.1371/journal.pone.0121694 (PMC4368097; doi:10.1371/journal.pone.0121694)
Supplement: S2 Table — (DOC) [file pone.0121694.s002.doc]

| **S2 Table: Hospital separations for injuries due to unintentional falls [1], Aboriginal BC, 1991-2010 [2], by Health Service Delivery Area** | | | | | | | | | | | |
| --- | --- | --- | --- | --- | --- | --- | --- | --- | --- | --- | --- |
|  |  |  |  |  |  |  |  |  |  |  |  |
| **HSDA** | **P-years [3]** | **Obs [4]** | **Exp [5]** | **Rate [6]** | **95% CI for Rate** | | | **SRR [7]** | **95% CI for SRR** | | |
|  |  |  |  |  |  |  |  |  |  |  |  |
| 11 | 31,823 | 153 | 78 | 48 | 41 | - | 56 | 1.96 | 1.57 | - | 2.45 |
| 12 | 11,511 | 37 | 27 | 32 | 23 | - | 44 | 1.39 | 0.95 | - | 2.04 |
| 13 | 134,648 | 511 | 310 | 38 | 35 | - | 41 | 1.65 | 1.47 | - | 1.84 |
| 14 | 330,133 | 1,772 | 771 | 54 | 51 | - | 56 | 2.30 | 2.14 | - | 2.46 |
| 21 | 163,205 | 553 | 357 | 34 | 31 | - | 37 | 1.55 | 1.40 | - | 1.72 |
| 22 | 94,689 | 314 | 194 | 33 | 30 | - | 37 | 1.62 | 1.40 | - | 1.86 |
| 23 | 104,732 | 284 | 212 | 27 | 24 | - | 30 | 1.34 | 1.17 | - | 1.54 |
| 31 | 14,027 | 29 | 30 | 21 | 14 | - | 30 | 0.97 | 0.68 | - | 1.39 |
| 32 | 216,087 | 934 | 448 | 43 | 41 | - | 46 | 2.09 | 1.90 | - | 2.29 |
| 33 | 189,336 | 944 | 431 | 50 | 47 | - | 53 | 2.19 | 1.99 | - | 2.41 |
| 41 | 129,783 | 582 | 279 | 45 | 41 | - | 49 | 2.08 | 1.85 | - | 2.34 |
| 42 | 270,670 | 1,349 | 592 | 50 | 47 | - | 53 | 2.28 | 2.10 | - | 2.47 |
| 43 | 128,917 | 877 | 286 | 68 | 64 | - | 73 | 3.06 | 2.73 | - | 3.44 |
| 51 | 396,222 | 2,443 | 951 | 62 | 59 | - | 64 | 2.57 | 2.41 | - | 2.74 |
| 52 | 226,219 | 1,380 | 490 | 61 | 58 | - | 64 | 2.82 | 2.58 | - | 3.08 |
| 53 | 82,381 | 442 | 176 | 54 | 49 | - | 59 | 2.51 | 2.16 | - | 2.91 |
|  |  |  |  |  |  |  |  |  |  |  |  |
| Urban [8] | 748,653 | 3,087 | 1,594 | 41 | 40 | - | 43 | 1.94 | 1.85 | - | 2.04 |
| Not [9] | 1,775,727 | 9,517 | 4,038 | 54 | 53 | - | 55 | 2.36 | 2.29 | - | 2.43 |
|  |  |  |  |  |  |  |  |  |  |  |  |
| All HSDAs | 2,524,380 | 12,604 | 5,633 | 50 | 49 | - | 51 | 2.24 | 2.18 | - | 2.30 |
|  |  |  |  |  |  |  |  |  |  |  |  |

| **Notes:** |
| --- |
| 1. "Injury due to unintentional fall" defined as hospital separation with Most Responsible Diagnosis in the range ICD9:800-999 or |
| ICD10:S00-T98, and supplemental diagnosis in the range ICD9:E880-E888 or ICD10:W00-W19. |
| 2. Injuries occurring during the observation period 1991-Apr-01 to 2010-Mar-31. |
| 3. Person-years is the sum of the annual population counts times the fraction of each year included in the observation period. |
| 4. Observed number of injuries. |
| 5. Expected number, indirectly standardized, based on age and gender-specific rates in the total population of BC. |
| 6. Crude Rate per 10,000 person-years. |
| 7. Standardized Relative Risk (compared to the total population of BC) = Observed/Expected. |
| 8. Urban: aggregation of HSDAs 22, 23, 31, 32, 33 and 41, where > 62.3% of the HSDA population live in a large population centre. |
| 9. Not urban: aggregation of HSDAs 11, 12, 13, 14, 21, 42, 43, 51, 52, 53. |
