# Supplementary material for: Injury Hospitalizations Due to Unintentional Falls among the Aboriginal Population of British Columbia, Canada: Incidence, Changes over Time, and Ecological Analysis of Risk Markers, 1991-2010
Source: PLoS One. 2015 Mar 20;10(3):e0121694. doi: 10.1371/journal.pone.0121694 (PMC4368097; doi:10.1371/journal.pone.0121694)
Supplement: S3 Table — (DOC) [file pone.0121694.s003.doc]

| **S3 Table: Hospital separations for injuries due to unintentional falls [1], British Columbia, 1991-2010 [2], by gender and age** | | | | | | | | | |
| --- | --- | --- | --- | --- | --- | --- | --- | --- | --- |
|  |  |  |  |  |  |  |  |  |  |
| **Gender** | **Age** | **P-years [3]** | **Obs [4]** | **Exp [5]** | **Rate [6]** | **95% CI for Rate** | | | **SRR [7]** |
|  |  |  |  |  |  |  |  |  |  |
| F | 0-9 | 4,574,431 | 7,954 | 7,952 | 17 | 17 | - | 18 | 1 |
| F | 10-19 | 4,926,160 | 4,999 | 4,998 | 10 | 10 | - | 10 | 1 |
| F | 20-29 | 5,357,569 | 3,962 | 3,959 | 7 | 7 | - | 8 | 1 |
| F | 30-39 | 6,139,503 | 5,847 | 5,846 | 10 | 9 | - | 10 | 1 |
| F | 40-49 | 6,212,389 | 8,399 | 8,391 | 14 | 13 | - | 14 | 1 |
| F | 50-59 | 4,698,502 | 12,299 | 12,289 | 26 | 26 | - | 27 | 1 |
| F | 60-69 | 3,298,634 | 16,038 | 16,036 | 49 | 48 | - | 49 | 1 |
| F | 70-79 | 2,525,823 | 29,976 | 29,973 | 119 | 117 | - | 120 | 1 |
| F | 80+ | 1,741,897 | 61,820 | 61,813 | 355 | 352 | - | 358 | 1 |
|  |  |  |  |  |  |  |  |  |  |
| M | 0-9 | 4,813,810 | 10,887 | 10,885 | 23 | 22 | - | 23 | 1 |
| M | 10-19 | 5,202,666 | 12,727 | 12,722 | 24 | 24 | - | 25 | 1 |
| M | 20-29 | 5,295,672 | 9,042 | 9,025 | 17 | 17 | - | 17 | 1 |
| M | 30-39 | 6,031,142 | 10,201 | 10,186 | 17 | 17 | - | 17 | 1 |
| M | 40-49 | 6,189,574 | 12,036 | 12,017 | 19 | 19 | - | 20 | 1 |
| M | 50-59 | 4,743,835 | 11,393 | 11,378 | 24 | 24 | - | 24 | 1 |
| M | 60-69 | 3,259,464 | 11,189 | 11,180 | 34 | 34 | - | 35 | 1 |
| M | 70-79 | 2,149,161 | 13,736 | 13,731 | 64 | 63 | - | 65 | 1 |
| M | 80+ | 1,030,240 | 19,209 | 19,208 | 186 | 184 | - | 189 | 1 |
|  |  |  |  |  |  |  |  |  |  |

| **Notes:** |
| --- |
| 1. "Injury due to unintentional fall" defined as hospital separation with Most Responsible Diagnosis in the range ICD9:800-999 or |
| ICD10:S00-T98, and supplemental diagnosis in the range ICD9:E880-E888 or ICD10:W00-W19. |
| 2. Injuries occurring during the observation period 1991-Apr-01 to 2010-Mar-31. |
| 3. Person-years is the sum of the annual population counts times the fraction of each year included in the observation period. |
| 4. Observed number of injuries. |
| 5. Expected number, indirectly standardized, based on age, gender and HSDA-specific rates in the total population of BC. |
| 6. Crude Rate per 10,000 person-years. |
| 7. Standardized Relative Risk (compared to the total population of BC) = Observed/Expected. |
