# Supplementary material for: Injury Hospitalizations Due to Unintentional Falls among the Aboriginal Population of British Columbia, Canada: Incidence, Changes over Time, and Ecological Analysis of Risk Markers, 1991-2010
Source: PLoS One. 2015 Mar 20;10(3):e0121694. doi: 10.1371/journal.pone.0121694 (PMC4368097; doi:10.1371/journal.pone.0121694)
Supplement: S4 Table — (DOC) [file pone.0121694.s004.doc]

| **S4 Table: Hospital separations for injuries due to unintentional falls [1], Aboriginal BC, 1991-2010 [2], by gender and age** | | | | | | | | | | | | |
| --- | --- | --- | --- | --- | --- | --- | --- | --- | --- | --- | --- | --- |
|  |  |  |  |  |  |  |  |  |  |  |  |  |
| **Gender** | **Age** | **P-years [3]** | **Obs [4]** | **Exp [5]** | **Rate [6]** | **95% CI for Rate** | | | **SRR [7]** | **95% CI for SRR** | | |
|  |  |  |  |  |  |  |  |  |  |  |  |  |
| F | 0-9 | 280,345 | 815 | 538 | 29 | 27 | - | 31 | 1.51 | 1.39 | - | 1.65 |
| F | 10-19 | 223,387 | 469 | 280 | 21 | 19 | - | 23 | 1.67 | 1.49 | - | 1.88 |
| F | 20-29 | 202,261 | 530 | 200 | 26 | 24 | - | 29 | 2.65 | 2.31 | - | 3.04 |
| F | 30-39 | 209,017 | 738 | 265 | 35 | 33 | - | 38 | 2.78 | 2.47 | - | 3.14 |
| F | 40-49 | 169,098 | 786 | 285 | 46 | 43 | - | 50 | 2.76 | 2.46 | - | 3.10 |
| F | 50-59 | 101,602 | 921 | 322 | 91 | 85 | - | 97 | 2.86 | 2.56 | - | 3.19 |
| F | 60-69 | 55,951 | 850 | 340 | 152 | 142 | - | 162 | 2.50 | 2.25 | - | 2.78 |
| F | 70-79 | 27,666 | 783 | 381 | 283 | 264 | - | 303 | 2.05 | 1.86 | - | 2.27 |
| F | 80+ | 14,369 | 691 | 589 | 481 | 447 | - | 517 | 1.17 | 1.08 | - | 1.27 |
|  |  |  |  |  |  |  |  |  |  |  |  |  |
| M | 0-9 | 294,127 | 1,107 | 718 | 38 | 35 | - | 40 | 1.54 | 1.43 | - | 1.66 |
| M | 10-19 | 232,572 | 825 | 661 | 35 | 33 | - | 38 | 1.25 | 1.16 | - | 1.35 |
| M | 20-29 | 197,321 | 707 | 408 | 36 | 33 | - | 39 | 1.73 | 1.57 | - | 1.91 |
| M | 30-39 | 198,365 | 834 | 418 | 42 | 39 | - | 45 | 1.99 | 1.81 | - | 2.19 |
| M | 40-49 | 155,287 | 803 | 369 | 52 | 48 | - | 55 | 2.18 | 1.97 | - | 2.41 |
| M | 50-59 | 90,787 | 634 | 258 | 70 | 65 | - | 75 | 2.46 | 2.18 | - | 2.78 |
| M | 60-69 | 48,676 | 529 | 210 | 109 | 100 | - | 118 | 2.52 | 2.20 | - | 2.88 |
| M | 70-79 | 22,387 | 378 | 167 | 169 | 153 | - | 187 | 2.27 | 1.95 | - | 2.64 |
| M | 80+ | 9,915 | 239 | 210 | 241 | 213 | - | 273 | 1.14 | 1.00 | - | 1.30 |
|  |  |  |  |  |  |  |  |  |  |  |  |  |
|  |  |  |  |  |  |  |  |  |  |  |  |  |
| **Notes:** |  |  |  |  |  |  |  |  |  |  |  |  |
| 1. "Injury due to unintentional fall" defined as hospital separation with Most Responsible Diagnosis in the range ICD9:800-999 or | | | | | | | | | | | | |
| ICD10:S00-T98, and supplemental diagnosis in the range ICD9:E880-E888 or ICD10:W00-W19. | | | | | | | | |  |  |  |  |
| 2. Injuries occurring during the observation period 1991-Apr-01 to 2010-Mar-31. | | | | | | |  |  |  |  |  |  |
| 3. Person-years is the sum of the annual population counts times the fraction of each year included in the observation period. | | | | | | | | | | | |  |
| 4. Observed number of injuries. | | |  |  |  |  |  |  |  |  |  |  |
| 5. Expected number, indirectly standardized, based on age, gender and HSDA-specific rates in the total population of BC. | | | | | | | | | | |  |  |
| 6. Crude Rate per 10,000 person-years. | | | |  |  |  |  |  |  |  |  |  |
| 7. Standardized Relative Risk (compared to the total population of BC) = Observed/Expected. | | | | | | | |  |  |  |  |  |
