# Supplementary material for: Injury Hospitalizations Due to Unintentional Falls among the Aboriginal Population of British Columbia, Canada: Incidence, Changes over Time, and Ecological Analysis of Risk Markers, 1991-2010
Source: PLoS One. 2015 Mar 20;10(3):e0121694. doi: 10.1371/journal.pone.0121694 (PMC4368097; doi:10.1371/journal.pone.0121694)
Supplement: S5 Table — (DOC) [file pone.0121694.s005.doc]

| **S5 Table: Hospital separations for injuries due to unintentional falls [1], British Columbia, 1991-2010 [2], by calendar year** | | | | | | | | | | | |
| --- | --- | --- | --- | --- | --- | --- | --- | --- | --- | --- | --- |
|  |  |  |  |  |  |  |  |  |  |  |  |
| **Year** | **P-years [3]** | **Obs [4]** | **Exp [5]** | **Rate [6]** | **95% CI for Rate** | | | **SRR [7]** | **95% CI for SRR** | | |
|  |  |  |  |  |  |  |  |  |  |  |  |
| 1991 | 2,566,094 | 10,109 | 7,894 | 39 | 39 | - | 40 | 1.28 | 1.25 | - | 1.31 |
| 1992 | 3,515,345 | 13,773 | 10,892 | 39 | 39 | - | 40 | 1.26 | 1.24 | - | 1.29 |
| 1993 | 3,649,925 | 14,191 | 11,307 | 39 | 38 | - | 40 | 1.26 | 1.23 | - | 1.28 |
| 1994 | 3,771,519 | 13,898 | 11,774 | 37 | 36 | - | 37 | 1.18 | 1.16 | - | 1.20 |
| 1995 | 3,856,183 | 13,826 | 12,120 | 36 | 35 | - | 36 | 1.14 | 1.12 | - | 1.16 |
| 1996 | 3,959,300 | 13,947 | 12,547 | 35 | 35 | - | 36 | 1.11 | 1.09 | - | 1.13 |
| 1997 | 4,040,687 | 13,989 | 12,922 | 35 | 34 | - | 35 | 1.08 | 1.06 | - | 1.10 |
| 1998 | 4,087,714 | 13,621 | 13,229 | 33 | 33 | - | 34 | 1.03 | 1.01 | - | 1.05 |
| 1999 | 4,115,601 | 13,743 | 13,521 | 33 | 33 | - | 34 | 1.02 | 1.00 | - | 1.03 |
| 2000 | 4,114,815 | 14,036 | 13,690 | 34 | 34 | - | 35 | 1.03 | 1.01 | - | 1.04 |
| 2001 | 4,160,615 | 13,689 | 14,002 | 33 | 32 | - | 33 | 0.98 | 0.96 | - | 0.99 |
| 2002 | 4,211,443 | 13,130 | 14,359 | 31 | 31 | - | 32 | 0.91 | 0.90 | - | 0.93 |
| 2003 | 4,285,095 | 13,204 | 14,731 | 31 | 30 | - | 31 | 0.90 | 0.88 | - | 0.91 |
| 2004 | 4,335,962 | 13,492 | 15,016 | 31 | 31 | - | 32 | 0.90 | 0.88 | - | 0.91 |
| 2005 | 4,383,639 | 13,455 | 15,361 | 31 | 30 | - | 31 | 0.88 | 0.86 | - | 0.89 |
| 2006 | 4,414,528 | 14,204 | 15,691 | 32 | 32 | - | 33 | 0.91 | 0.89 | - | 0.92 |
| 2007 | 4,476,436 | 13,990 | 16,075 | 31 | 31 | - | 32 | 0.87 | 0.86 | - | 0.88 |
| 2008 | 4,546,001 | 14,244 | 16,458 | 31 | 31 | - | 32 | 0.87 | 0.85 | - | 0.88 |
| 2009 | 4,607,365 | 14,717 | 16,835 | 32 | 31 | - | 32 | 0.87 | 0.86 | - | 0.89 |
| 2010 | 1,158,039 | 3,561 | 4,268 | 31 | 30 | - | 32 | 0.83 | 0.81 | - | 0.86 |
|  |  |  |  |  |  |  |  |  |  |  |  |
| 1991-2010 | 78,256,306 | 262,819 | 262,693 | 34 | 33 | - | 34 | 1 | [reference] | | |
|  |  |  |  |  |  |  |  |  |  |  |  |

| **Notes:** |
| --- |
| 1. "Injury due to unintentional fall" defined as hospital separation with Most Responsible Diagnosis in the range ICD9:800-999 or |
| ICD10:S00-T98, and supplemental diagnosis in the range ICD9:E880-E888 or ICD10:W00-W19. |
| 2. Injuries occurring during the observation period 1991-Apr-01 to 2010-Mar-31. |
| 3. Person-years is the annual population count times the fraction of the year included in the observation period. |
| 4. Observed number of injuries. |
| 5. Expected number, indirectly standardized, based on age, gender and HSDA-specific rates in the total population of BC during |
| the entire observation period. |
| 6. Crude Rate per 10,000 person-years. |
| 7. Standardized Relative Risk (compared to the total population of BC) = Observed/Expected. |
