# Supplementary material for: Injury Hospitalizations Due to Unintentional Falls among the Aboriginal Population of British Columbia, Canada: Incidence, Changes over Time, and Ecological Analysis of Risk Markers, 1991-2010
Source: PLoS One. 2015 Mar 20;10(3):e0121694. doi: 10.1371/journal.pone.0121694 (PMC4368097; doi:10.1371/journal.pone.0121694)
Supplement: S6 Table — (DOC) [file pone.0121694.s006.doc]

| **S6 Table: Hospital separations for injuries due to unintentional falls [1], Aboriginal BC, 1991-2010 [2], by calendar year** | | | | | | | | | | | |
| --- | --- | --- | --- | --- | --- | --- | --- | --- | --- | --- | --- |
|  |  |  |  |  |  |  |  |  |  |  |  |
| **Year** | **P-years [3]** | **Obs [4]** | **Exp [5]** | **Rate [6]** | **95% CI for Rate** | | | **SRR [7]** | **95% CI for SRR** | | |
|  |  |  |  |  |  |  |  |  |  |  |  |
| 1991 | 81,353 | 590 | 205 | 73 | 67 | - | 79 | 2.88 | 2.51 | - | 3.30 |
| 1992 | 111,758 | 804 | 284 | 72 | 67 | - | 77 | 2.83 | 2.52 | - | 3.18 |
| 1993 | 116,061 | 752 | 298 | 65 | 60 | - | 70 | 2.53 | 2.26 | - | 2.83 |
| 1994 | 119,614 | 718 | 308 | 60 | 56 | - | 65 | 2.33 | 2.09 | - | 2.61 |
| 1995 | 122,026 | 686 | 311 | 56 | 52 | - | 61 | 2.21 | 1.97 | - | 2.47 |
| 1996 | 124,891 | 651 | 319 | 52 | 48 | - | 56 | 2.04 | 1.83 | - | 2.27 |
| 1997 | 126,909 | 699 | 327 | 55 | 51 | - | 59 | 2.14 | 1.92 | - | 2.38 |
| 1998 | 128,332 | 668 | 332 | 52 | 48 | - | 56 | 2.01 | 1.81 | - | 2.24 |
| 1999 | 128,945 | 670 | 335 | 52 | 48 | - | 56 | 2.00 | 1.80 | - | 2.23 |
| 2000 | 130,683 | 703 | 340 | 54 | 50 | - | 58 | 2.07 | 1.86 | - | 2.30 |
| 2001 | 133,025 | 623 | 348 | 47 | 43 | - | 51 | 1.79 | 1.61 | - | 1.99 |
| 2002 | 135,727 | 609 | 357 | 45 | 41 | - | 49 | 1.70 | 1.54 | - | 1.89 |
| 2003 | 139,955 | 585 | 371 | 42 | 39 | - | 45 | 1.58 | 1.43 | - | 1.75 |
| 2004 | 142,881 | 677 | 381 | 47 | 44 | - | 51 | 1.78 | 1.61 | - | 1.96 |
| 2005 | 145,834 | 592 | 393 | 41 | 37 | - | 44 | 1.51 | 1.37 | - | 1.66 |
| 2006 | 148,458 | 617 | 404 | 42 | 38 | - | 45 | 1.53 | 1.38 | - | 1.68 |
| 2007 | 151,609 | 647 | 416 | 43 | 40 | - | 46 | 1.56 | 1.41 | - | 1.71 |
| 2008 | 154,876 | 609 | 428 | 39 | 36 | - | 43 | 1.42 | 1.29 | - | 1.56 |
| 2009 | 158,252 | 619 | 441 | 39 | 36 | - | 42 | 1.40 | 1.28 | - | 1.54 |
| 2010 | 39,871 | 164 | 112 | 41 | 35 | - | 48 | 1.47 | 1.22 | - | 1.77 |
|  |  |  |  |  |  |  |  |  |  |  |  |
| 1991-2010 | 2,541,060 | 12,683 | 6,710 | 50 | 49 | - | 51 | 1.89 | 1.85 | - | 1.94 |
|  |  |  |  |  |  |  |  |  |  |  |  |

| **Notes:** |
| --- |
| 1. "Injury due to unintentional fall" defined as hospital separation with Most Responsible Diagnosis in the range ICD9:800-999 or |
| ICD10:S00-T98, and supplemental diagnosis in the range ICD9:E880-E888 or ICD10:W00-W19. |
| 2. Injuries occurring during the observation period 1991-Apr-01 to 2010-Mar-31. |
| 3. Person-years is the annual population count times the fraction of the year included in the observation period. |
| 4. Observed number of injuries. |
| 5. Expected number, indirectly standardized, based on age, gender and HSDA-specific rates in the total population of BC during |
| the entire observation period. |
| 6. Crude Rate per 10,000 person-years. |
| 7. Standardized Relative Risk (compared to the total population of BC) = Observed/Expected. |
